# Living donor liver transplantation indicated for compensated liver cirrhosis with symptomatic gallstone diseases: report of two cases

**DOI:** 10.1186/s40792-016-0172-3

**Published:** 2016-05-23

**Authors:** Yuki Bekki, Toru Ikegami, Yoshihiro Yoshida, Takashi Motomura, Shinji Itoh, Noboru Harada, Norifumi Harimoto, Hideaki Uchiyama, Tomoharu Yoshizumi, Yoshihiko Maehara

**Affiliations:** Department of Surgery and Science, Graduate School of Medical Sciences, Kyushu University, 3-1-1, Maidashi Higashi-ku, Fukuoka, 812-8582 Japan

**Keywords:** Living donor liver transplantation, Gallstone disease, Liver cirrhosis

## Abstract

**Background:**

Surgical interventions for symptomatic gallstone disease could be dangerous in patients with severe comorbid conditions including liver cirrhosis. Here, we report our experience of living donor liver transplantation (LDLT) indicated for two patients with liver cirrhosis complicated with gallstone diseases.

**Case 1:**

A 70-year-old woman with a history of hepatitis C virus infection was diagnosed as symptomatic choledocholithiasis. She had open cholecystectomy and choledochotomy with choledocholithotomy, which complicated with postoperative liver failure. Her Child-Pugh score increased from 7 to 12 points and Model for End-Stage Liver Disease (MELD) score from 11 to 36. She underwent LDLT, using the right lobe graft donated by her 47-year-old daughter. The post-transplant graft function was excellent, and the patient was discharged from the hospital on postoperative day 27.

**Case 2:**

A 46-year-old man with a history of hepatitis B virus infection was diagnosed as cholecystitis. He had cholecystostomy without any complications and his Child-Pugh score remained to be 9 and MELD score 17, followed by LDLT using the right lobe graft donated by his 45-year-old wife. The post-transplant graft function was excellent, and the patient was discharged from the hospital on postoperative day 44.

**Conclusion:**

LDLT is one of treatment options when patients with Child-Pugh B cirrhosis accompanied with gallstone diseases, likely to be deteriorating their liver functions in the near future.

## Background

Gallstones are common and detected as high as 4.11 % in general population [1], and the rate would be 1.2 to 3 times higher in patients with chronic liver disease (CLD) [2, 3]. Acute cholecystitis and cholangitis results from obstruction of the cystic duct or common bile duct, respectively, usually by gallstones, followed by inflammation of the gallbladder, or bile duct. Laparoscopic cholecystectomy (LC) is the standard treatment for acute cholecystitis in general population [4]. Cholangitis, on the other hand, requires biliary decompression by endoscopic or percutaneous transhepatic approaches and antibiotic therapy [5]. Patients with symptoms caused by gallstone (GS) disease can be difficult to manage if they have severe comorbid conditions, including end-stage liver disease [6]. Morbidity and mortality after cholecystectomy get greater in accords with Child-Pugh class [2], and Machado reported that mortality after cholecystectomy of patients with Child-Pugh A, B, and C liver cirrhosis was 0.12, 0.97, and 17.1 %, respectively [7].

Because a poor outcome was observed, some study recommends that percutaneous gall bladder aspiration [8, 9] and endoscopic gallbladder stent placement [2, 10] rather than operation may be a potential treatment or temporizing measure for symptomatic gallbladder disease in patients with liver cirrhosis awaiting liver transplantation (LT) [9, 11]. It is difficult, however, to await LT with temporizing measure in Japan, considering the severe shortage of cadaveric donor grafts [12]. Here, we report our experience of living donor liver transplantation (LDLT) for two patients with liver cirrhosis complicated with GS diseases.

## Case presentation

### Case 1

**Fig. 1:**
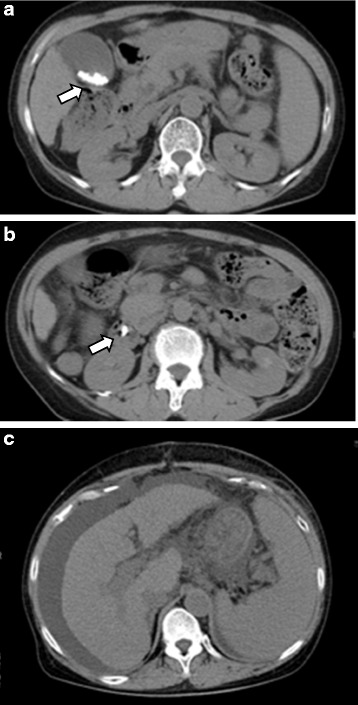
**a**–**c** Pretransplant abdominal CT imaging studies in our case 1 before primary operation showed stones in the gallbladder (*arrow* in **a**) and common bile duct (*arrow* in **b**). After primary operation, abdominal CT imaging showed shrinking liver and ascites retention (**c**)

A 70-year-old woman with hepatitis C virus (HCV) infection and liver cirrhosis without any treatment came to the primary hospital complaining of abdominal pain, which was diagnosed as symptomatic choledocholithiasis. Her hepatic and renal profiles on admission were as follows: total bilirubin, 1.6 mg/dl; albumin, 2.9 g/dl; aspartate aminotransferase (AST), 45 U/l; international normalized ratio (INR), 1.26; and creatinine, 0.69 mg/dl. Endoscopic drainage was indicated, which failed unfortunately because of esophageal varices. She did not show any improvement with 22-day conservative treatment, so she was scheduled for open cholecystectomy and choledochotomy with choledocholithotomy as a radical treatment. The operation itself was undergone without trouble with operation time of 200 min and blood loss of 316 ml. She was complicated with postoperative liver failure, however, with grade 1 encephalopathy and hyperbilirubinemia as high as total bilirubin 22.1 mg/dl on the postoperative day 12. So she was referred to our hospital on the postoperative day 32 for possible LDLT after several plasma exchange did not show any improvement. At the time of referral, her hepatic and renal profiles were as follows: total bilirubin, 26.4 mg/dl; albumin, 2.9 g/dl; aspartate aminotransferase (AST), 65 U/l; international normalized ratio (INR), 1.42; and creatinine, 0.81 mg/dl. Abdominal computed tomography (CT) imaging studies showed massive ascites after the first operation (Fig. 1). Her Child-Pugh score increased from 7 (grade B) to 12 points (grade C) and Model for End-Stage Liver Disease (MELD) score from 11 to 36. LT was indicated for her liver failure and she underwent LDLT, using a blood type identical to that of the right lobe graft donated by her 47-year-old daughter. The graft volume was 520 g and graft volume to recipient standard liver volume ratio (GV/SLV) was 51.1 %. The post-transplant graft function was excellent, and the patient was discharged from the hospital on postoperative day 27.

### Case 2

**Fig. 2:**
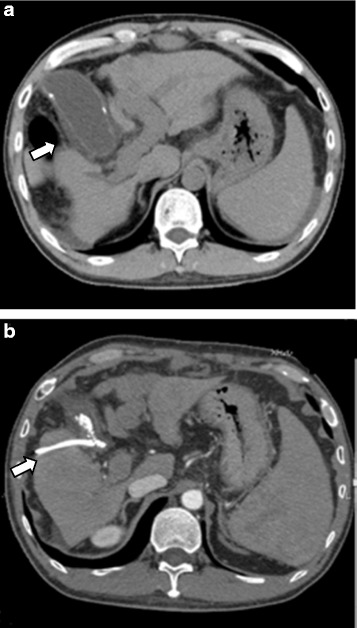
**a**–**b**. Pretransplant abdominal computed tomography CT imaging studies in our case 2 showed gallbladder distention and wall thickness before cholecystostomy (*arrow* in **a**), and cholecystostomy (*arrow* in **b**) resolved these findings

A 46-year-old man with a history of esophageal varices with endoscopic variceal ligation (EVL) was pointed out of hepatitis B virus (HBV) infection and liver cirrhosis treated with entecavir. He was admitted to the hospital for the treatment of cholecystitis. We attempted percutaneous drainage with cholecystostomy to relief of his symptom and scheduled LDLT for definitive treatment of liver cirrhosis and cholecystitis. Open or laparoscopic cholecystectomy might be indicated, but he would require LT sooner or later considering his background factors; his age of 40s with progressively deteriorating liver function, and his liver disease of HBV in which reinfection could be controlled with hepatitis B immune globulin (HBIG) and antiviral agents such as lamivudine or adefovir [13]. His hepatic and renal profiles before LDLT were as follows: total bilirubin, 4.3 mg/dl; albumin, 3.0 g/dl; AST, 38 U/l; INR, 1.55; and creatinine, 0.57 mg/dl. Abdominal CT imaging studies did not show any change without inserted cholecystostomy tube and relieved inflammation in gallbladder (Fig. 2). His Child-Pugh score remained to be 9 (grade B) and MELD score 17, both score showing no change before and after intervention of cholecystostomy unlike the patient in our case 1. LT was indicated for him and she underwent LDLT, using a blood type identical to that of the right lobe graft donated by his 45-year-old wife. The graft volume was 520 g and GV/SLV was 41.8 %. The post-transplant graft function was excellent, and the patient was discharged from the hospital on postoperative day 44.

### Discussion

Gallbladder disease of patients with liver cirrhosis is difficult to manage [6]. Open or laparoscopic cholecystectomy has been reported to be performed safely in cirrhotic patients with well-compensated liver function [14], but in our case 1 the patient with Child-Pugh B cirrhosis suffered postoperative liver failure. Postoperative liver failure without improvement with plasma exchanges is indicated for liver transplantation; and considering the scarcity of cadaveric liver grafts in Japan [12], LDLT was indicated. Endoscopic treatment would be the first-choice treatment [4], but cirrhotic patients are at high risk of accompanying with esophageal varices as the patient in our case 1, which interrupt endoscopic treatment.

The best management of cirrhotic patients with symptomatic GS might require a multidisciplinary team of surgeons, endoscopists, and radiologists, to allow the most efficient treatment including LT [15]. For example in our case 2, we performed percutaneous cholecystostomy which controlled symptoms considering the risks of liver failure following surgery like case 1. Definitive treatment was mandate in this case with multiple stones stuck in the gallbladder neck and anticipated relapsing cholecystitis. We did not treat by cholecystectomy, with preparing LDLT just in case, because the results of emergency LDLT have been considered inferior to those of elective transplants [16]. Rather, we preceded percutaneous cholecystostomy followed by LDLT.

**Fig. 3:**
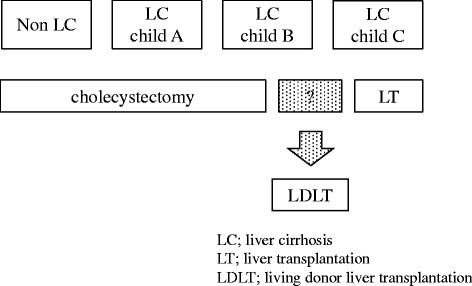
Treatment algorithm for symptomatic gallstone disease complicated with cirrhotic patients

Studies that stratified patients by Child-Pugh status found greater morbidity and mortality after cholecystectomy in accords with Child-Pugh class [2]. Some study concluded to be safe for patients with liver cirrhosis of Child-Pugh A and B to undergo laparoscopic cholecystectomy [7, 17–20]. On the other hand, a poor outcome with cholecystectomy was observed in Child-Pugh C cirrhotic patients [9] and LT seems to be the only way [17]. So patients whose liver function at the boundary between Child-Pugh B and C would be challenge to treat, and nonsurgical therapies including percutaneous cholecystostomy and ERC stent insertion and drainage have frequent recurrence. So we propose that LT would be treatment options for Child-Pugh B cirrhosis complicated with GS disease; and considering recipient and donor factors, LDLT could be indicated which treats both liver cirrhosis and symptomatic GS disease (Fig. 3).

## Conclusions

Cirrhotic patients with Child-Pugh class B and C with symptomatic GS disease are life-threatening conditions with significant mortality. Multidisciplinary management in a tertiary care center considering LDLT as an option may offer the optimal treatment.

## Consent

Written informed consent was obtained from the patient for publication of this case report and accompanying images.

## Abbreviations

AST, aspartate aminotransferase; CLD, chronic liver disease; CT, computed tomography; EVL, endoscopic variceal ligation; GS, gallstone; GV/SLV, graft volume to recipient standard liver volume ratio; HBIG, hepatitis B immune globulin; HBV, hepatitis B virus; HCV, hepatitis C virus; INT, international normalized ratio; LC, laparoscopic cholecystectomy; LDLT, living donor liver transplantation; LT, liver transplantation; MELD, Model for End-Stage Liver Disease
